# Neuroprotective Properties of Chitosan and Its Derivatives

**DOI:** 10.3390/md8072117

**Published:** 2010-07-09

**Authors:** Ratih Pangestuti, Se-Kwon Kim

**Affiliations:** 1 Marine Biochemistry Laboratory, Department of Chemistry, Pukyong National University, Busan 608-737, Korea; E-Mail: ratihpangestuti@pknu.ac.kr (R.P.); 2 Marine Bioprocess Research Center, Pukyong National University, Busan 608-737, Korea

**Keywords:** neuronal cells, neuroprotection, neuroprotective agents, neuroprotective properties, chitosan and derivatives

## Abstract

Neuronal cells are extremely vulnerable and have a limited capacity for self-repair in response to injury. For those reasons, there is obvious interest in limiting neuronal damage. Mechanisms and strategies used in order to protect against neuronal injury, apoptosis, dysfunction, and degeneration in the central nervous system are recognized as neuroprotection. Neuroprotection could be achieved through several classes of natural and synthetic neuroprotective agents. However, considering the side effects of synthetic neuroprotective agents, the search for natural neuroprotective agents has received great attention. Recently, an increasing number of studies have identified neuroprotective properties of chitosan and its derivatives; however, there are some significant challenges that must be overcome for the success of this approach. Hence, the objective of this review is to discuss neuroprotective properties of chitosan and its derivatives.

## 1. Introduction

The brain is an amazing and critical organ for our life [1]. It is surrounded by layers of tissue called meninges and encased by the skull. There are two broad classes of cells in the brain, which are neuron and glia [2–4]. Even though neuronal cells are among the longest-living cell types in mammals, unlike many other cells, they have a limited capacity for self-repair in response to injury [5,6]. This condition is compounded by the fact that neuronal cells are extremely vulnerable [7]. For this reason, there is an obvious interest in limiting the cell damage caused by various insults that trigger endogenous repair mechanisms. The mechanisms and strategies used in order to protect them against neuronal injury, apoptosis, dysfunction and or degeneration in the central nervous system (CNS) are called as neuroprotection [8]. The goal of neuroprotection is to limit neuronal dysfunction or death after CNS injury in an attempt to maintain the highest possible integrity of cellular interactions in the brain, thus minimizing disturbance to neural function [9]. According to its mechanism, neuroprotection can be categorized into several mechanisms such as: antioxidant (free radical trapper/scavenger) [10,11]; anti-inflammatory [12,13]; anti-exitotoxic [14]; apoptosis inhibitor [15]; gene expression modulator [16]; ion channel modulator [17,18]; metal ion chelator [19,20]; neurotrophic factor [21–23]; MMP inhibitor [24]; combined mechanism (combining two mechanisms or more) [25].

Many categories of natural and synthetic compounds have been reported to possess a neuroprotective activity. However, these synthetic neuroprotective agents are believed to have certain side effects such as dry mouth, tiredness, drowsiness, sleepiness, anxiety or nervousness, difficulty to balance, *etc.* [26]. Hence, nowadays researchers have a great interest to study natural bioactive compounds that can act as neuroprotective agents.

The marine environment is been known as a rich source of bioactive chemical structures with promising biological activities such as neuroprotection [27]. Based on several studies, it is reported that chitosan, one of the biologically active compounds derived from the sea, has potent neuroprotective properties.

Over the last three decades, there have been a growing number of publications on chitosan and its derivatives in the pharmaceutical industry. Chitosan is a linear polysaccharide that consists of β-(1→4)-2-acetamido-d-glucose and β-(1→4)-2-amino-d-glucose units derived from partial deacetylation of chitin [28,29]. However, this polysaccharide has poor solubility, making it difficult to be used in food and biomedical applications [30]. Considering this property limitation, some researchers are interested in converting chitosan into oligosaccharides [31]. Chitooligosaccharides (COS), oligosaccharides form of chitosan, are readily soluble in water due to their shorter chain lengths and free amino groups in d-glucosamine units [32]. Similar to chitosan, COS have positive charges resulting from removal of acetyl units from D-glucosamine residues. These properties enable COS to interact with negatively charged polymers, macromolecules and polyanions in an aqueous environment [33,34]. Both chitosan and COS are known to possess many biological activities such as antibacterial [31,35], immunoenhancing [36], antioxidant [37], matrix metalloproteinase (MMP) inhibition [38–40], anti-diabetic [41], anti-HIV [42], anti-inflammatory activities [43], and drug delivery [44], *etc.* Not only restricted to those activities, chemical modification will enhance and open various ways to utilize chitosan and COS [29]. The rationale for this is that a chemical modification will keep the original physiochemical and biochemical properties of chitosan and COS and bring the new properties of the group introduced to them at the same time [45].

Recent reviews have been published regarding the pharmaceutical effects of chitosan and its derivatives. This review, however, focuses specifically on neuroprotective properties of chitosan and its derivatives.

## 2. Suppressing Effect on β-Amyloid Formation

The most common neurodegenerative disorder, Alzheimer’s disease (AD), is an irreversible, progressive brain disease affecting cognition [46]. The pathological hallmark of AD is the deposition of senile plaques (SPs) and neurofibrillary tangles (NFTs) [47]. SPs are composed of the β-amyloid (Aβ) peptides, which are cleaved from amyloid precursor proteins (APPs) by proteolysis enzymes such as β- and γ-secretase [48–50]. In APP proteolysis, it seems that the key enzyme is β-secretase, which is also known as β-amyloid cleavage enzyme (BACE-1), since it initiates the formation of Aβ [51]. Hence, BACE-1 represents a candidate biomarker, as well as a drug target for AD [52].

There have been some studies on BACE-1 inhibition activities and Aβ formation inhibition activities of chitosan and its derivatives for a decade.

**Scheme 1:**
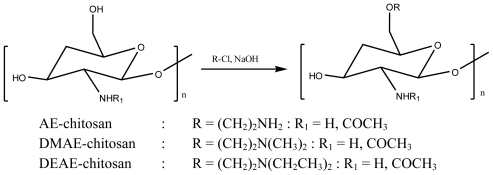
Synthesis of chitosan derivatives.

BACE-1 inhibition activity of chitosan derivatives have been reported by Je *et al.* [28]. They prepared chitosan with two degrees of deacetylation (90% and 50%) and grafted amino functionality into chitosan to improve the solubility and bioactivity. The synthesized reaction of chitosan derivatives are presented in Scheme 1. Chitosan derivatives were designated as aminoethyl (AE-chitosan) (90%), dimethylaminoethyl (DMAE-chitosan) (90%), and diethylaminoethyl (DEAE-chitosan) (90%) prepared from 90% deacetylated chitosan, and AE-chitosan (50%), DMAE-chitosan (50%), and DEAE-chitosan (50%) prepared from 50% deacetylated chitosan. The potencies of chitosan derivatives are expressed as an IC_50_ value, which is the BACE-1 inhibitor concentration leading to 50% inhibition of BACE-1 activity. AE-chitosan (90%) shows strongest inhibitory activity compared to other derivatives. Moreover, the inhibition modes of BACE-1 catalyzed by chitosan and its derivatives have been determined by Dixon plots. Based on the Dixon plots, the inhibition constant (*K_i_*) of AE-chitosan (90%) is 85 μg/mL. They suggested that the free amino groups at the C-2 and C-6 positions play an important role in BACE-1 inhibitory activity; however, the free amino group at C-2 is a minor factor according to the above results.

An investigation of BACE-1 inhibitory activity of COS was done by Byun *et al.* [53]. Nine kinds of hetero-COS with different degrees of deacetylation and molecular weight were prepared by using an ultrafiltration (UF) membrane reactor [35]. The deacetylated chitosan of 90, 75, and 50% were hydrolyzed and fractionated by passing them through three UF membranes of molecular weight cut-off (MWCO) 10, 5, and 1 kDa. The hetero-COSs were named 90-HMWCOSs, 75-HMWCOSs, 50-HMWCOSs, 90-MMWCOSs, 75-MMWCOSs, 50-MMWCOSs, 90-LMWCOSs, 75-LMWCOSs, 50-LMWCOSs respectively. 90-MMWCOSs which are 90% deacetylated COS with molecular weight 3–5 kDa, exhibited the highest BACE-1 inhibitory activity (25–42 mM) compared to the others. The inhibitor was found to have a noncompetitive by Dixon plot, and the *Ki* of 90-MMWCOSs was 3.87–6.47 mM. The study of Byun and his colleagues indicated that degree of deacetylation and sulfations at the C-2 position of COS has an effect on BACE-1 inhibitory activity. A further amine group at C-2 position was shown to be beneficial for BACE-1 inhibitory activity.

Based on the hypothesis that the increase of an important product of oxidation may induce Aβ formation, Khodagholi *et al.* [54] studied the effect of chitosan in NT2 neuron cells induced by H_2_O_2_ and FeSO_4_. NT2 neuronal cells are a widely accepted experimental model to study the regulation of APP metabolism and the pathogenesis of AD [55]. In their study, they found that Aβ formation by NT2 neurons pretreated with chitosan was significantly lower than that of control cells exposed only to H_2_O_2_. The Aβ levels rose from 30.96 pg/mL in H_2_O_2_-treated cells to 22.2 and 18.35 pg/mL in the presence of 0.1 and 0.5% w/v chitosan, respectively. This study indicates that Aβ level can be controlled by treatment with this chitosan, suggesting a protective effect of chitosan in AD.

Almost all currently available medications for AD are cholinesterase inhibitors. Considering these reasons, the suppression of β-amyloid formation by chitosan and its derivatives will enhance the medications for AD. However, further studies are needed with clinical trials for the application of chitosan and its derivatives in AD medications.

## 3. Acetylcholinesterase Inhibitory Activity

The pathogenesis of AD has been linked to a deficiency in the brain neurotransmitter acetylcholine (ACh) [56]. This was stated in the cholinergic hypothesis which was raised three decades ago, that a serious loss of cholinergic function in the CNS contributes significantly to the cognitive symptoms associated with AD [57]. The inhibition of acetylcholinesterase (AChE) enzyme, which catalyzes the breakdown of ACh, may be one of the most realistic approaches to the symptomatic treatment of AD [56,58,59].

Recently, several studies on chitosan and its derivatives have identified their potential as acetylcholinesterase inhibitors (AChEIs). The AChEIs activity of COS and its derivatives is discussed below as well as summarized in Table 1.

Lee *et al*. [60] studied AChEIs activity of six kinds of COS with different molecular weight and degrees of deacetylation. In their study, 90-COS and 50-COS were prepared from 90% and 50% deacetylated chitosan and further fractionated into three kinds of COS, high molecular weight (HMW) (5,000–10,000 dalton), medium molecular weight (MMW) (1,000–5,000 dalton), and low molecular weight (LMW) (below 1,000 dalton) using an ultrafiltration membrane system as described by Kim *et al.* [35]. 90-COS has stronger AChEIs activity than 50-COS. Among 90-COS, 90-MMWCOS (90% deacetylated COS with molecular weight 1000–5000 Da) showed the strongest AChEIs with IC_50_ value of 1.67 mg/mL (Table 1). Moreover, Lee *et al*. also investigated the level of AChE protein expression and AChEIs activity in PC12 cell lines using Ellman’s evaluation. The results showed that 90-MMWCOS suppressed the AChE protein expression and increased the AChEIs in a dose dependant manner. These findings suggest that degree of deacetylation of COS is the key factor for the AChEIs activity.

Furthermore, Yoon *et al*. [61] synthesized COS derivatives with different substitution groups. In their study, the synthesis of COS derivatives was accomplished by the displacement of the hydroxyl group at the C-6 of the pyranose ring and replaced with aminoethyl (AE), dimethylaminoethyl (DMAE) and diethylaminoethyl (DEAE) groups. The chemical structures were determined as AE-COS, DMAE-COS and DEAE-COS, in sequence. Eserine, a parasympathomimetic and a reversible cholinesterase inhibitor, was used as the positive control in their study. Among three COS derivatives, DEAE-COS has the strongest AChEIs activity with IC_50_ values of 9.2 ± 0.33 μg/mL. DMAE- and DEAE-COS were identified as competitive AChEIs according to the Lineweaver–Burk plot. These findings suggest that the chemical modification will enhance the utilization of COS as AChEIs, and their inhibitory activity depends on the hydrophobic nature of the group that is introduced to them.

AChEIs, which provide modest symptomatic improvement and are able to delay the loss of functional abilities, is a promising medication for AD patients. The AChEIs activity of COS and its derivatives indicate that COS derivatives might be a beneficial material in the prevention or treatment of AD. Furthermore, mechanistic studies, particularly those that investigate how these compounds inhibit AChE activity in cellular systems, will be required in the future.

## 4. Anti-Neuroinflammatory

In principle, inflammation is the first response of a human body’s immune system to pathogens or irritation [62,63]. A growing number of studies are discovering intriguing links between chronic inflammation and a number of neurodegenerative disorders [62]. The neuroinflammation process plays a pivotal role in the initiation and progression of various neurodegenerative diseases. Recently, a number of studies have found anti-neuroinflammatory activity of chitosan and its derivatives.

Kim *et al*. [64] reported that high molecular weight water soluble chitosan (WSC) inhibits the production of pro-inflammatory cytokine in human astrocytoma cells activated by Aβ peptide 25–35 (Aβ_25–35_) and interleukin-1β (IL-1β). In their study, they used the human astrocytoma cell line (CCF-STTG1) as an *in vitro* AD model. The effects of WSC on pro-inflammatory cytokines such as tumor necrosis factor-α (TNF-α) and interleukin-6 (IL-6) were evaluated by enzyme-linked immunosorbent assay (ELISA) and western blotting. The secretion and expression of TNF-α and IL-6 were significantly inhibited by pretreatment with 1 and 10 μg/mL of WSC. Moreover, the expression of inducible nitric-oxide synthase (iNOS) induced by Aβ_25–35_ and IL-1β was partially inhibited by treatment with WSC. However, those findings need further investigation to find whether WSC regulates the transcription factor and signaling molecules with concerns to the production of inflammatory cytokines and neurotoxic components.

Another study conducted by Khodagholi *et al*. focused on the anti-neuroinflammatory effect of chitosan and its derivatives on NT2 neuronal cells. Chitosan exerts anti-neuroinflammatory action by upregulation of heat shock protein 70 (Hsp-70) and inhibits the activation of NF-κB [54]. The anti-inflammatory mechanism of Hsp-70 is mediated by the binding of Hsp-70 to NF-κB and its subsequent inhibition [65]. A pre-treatment with 0.1 and 0.5% (w/v) chitosan prior to H_2_O_2_ and FeSO_4_ exposure has been proved to increase the level of heat shock protein (Hsp-70) to 1.4- and 1.6-times, respectively.

Several studies have shown anti-neuroinflammatory activity of chitosan and its derivatives; however, further studies about anti-neuroinflammatory activity of chitosan and its derivatives in other neuronal cells such as microglia are needed. Moreover, various intracellular signaling pathways also need to be investigated in order to obtain a better understanding of the underlying anti-neuroinflammatory mechanism of chitosan and its derivatives.

## 5. Apoptosis Inhibitors

The elimination of cells by apoptosis or programmed cell death is a fundamental event in development, whereby multi-cellular organisms regulate cell numbers or eliminate cells that are functionally redundant or potentially detrimental to the organism [66]. Many human diseases such as acquired immunodeficiency syndrome, neurodegenerative disorders, and cancer can be attributed directly or indirectly to a derangement of apoptosis resulting in either cell accumulation, in which cell eradication or cell turnover is impaired or cell loss, in which the apoptotic programs are inadvertently triggered [67]. In neurodegenerative disorders, apoptosis might be pathogenic, and targeting it might mitigate neurodegenerative disorders [68]. Some studies on chitosan have found biological activity of chitosan, COS and its derivatives in targeting apoptosis in brain cells.

A study carried out by Koo *et al.* successfully showed that high molecular weight water soluble chitosan (WSC) was able to protect against apoptosis in human astrocytoma cells (CCF-STTG1) induced by serum starvation [69]. In their study, they used WSC, which has a molecular weight of 300 kDa and degree of deacetylation over 90%, which was produced by using a multi-step membrane separation process. Based on the cytotoxicity test, WSC (10 μg/mL) was able to inhibit cell death significantly compared to the control group. Furthermore, in order to induce apoptosis by serum starvation, they incubated CCF-STTG1 cells for 48 h in medium supplemented with only 0.1% FBS. The serum starvation-induced apoptosis of CCF-STTG1 cells was determined by flow cytometry. Their results suggested that when the cells treated with WSC (10 μg/mL) were exposed to serum starved-medium, apoptosis of CCF-STTG1 cells was almost completely inhibited compared to the effect of serum starved-medium alone. Supporting the flow cytometry, they also showed that DNA fragmentation in a ladder pattern, which is characteristic of apoptosis, was not detected in CCF-STTG1 cells pre-treated with WSC (10 μg/mL). It is known that serum starvation induces apoptosis through the activation of p53, based on their Western blot data; WSC can prevent serum starvation-induced apoptosis via blocking p53 activation. Although their study did not directly demonstrate general cell death of CCF-STTG1 astrocytoma cells because only morphological changes have been observed, their finding might suggest that WSC can promote neuroprotective properties in the brain.

Recently, the protective effect of COS, with a molecular weight 800 Da, against glutamate-induced neurotoxicity in cultured hippocampal neurons has been reported [70]. In their study they used glutamate as a model, because glutamate accumulation in the CNS and excessive stimulation of glutamate receptors induces potent neurotoxic action, which is specifically referred to as excitotoxicity, and is involved in neuronal damage and degenerative disorders in the CNS. A glutamate concentration of 125 μM was chosen in their study. Hoechst staining and flow cytometry with annexin V/PI staining showed that in this concentration rat hippocampal neurons underwent extensive apoptotic-like cell death characterized by neuronal morphology. Based on the cell viability assessments, together with Hoechst 33342 staining and flow cytometry for cell apoptosis analysis, pre-treatment with COS was able to attenuate apoptosis in hippocampal neurons cells in a dose dependent manner. Moreover, they also measured the change in caspase-3 activity during cell treatment. A three- to four-fold increase in caspase-3 activity was found in cultured hippocampal neurons at 18 h after a 15-min exposure to glutamate, and pretreatment with 1.0 and 2.0 mg/mL of COS prevented cells from glutamate-induced increases in caspase-3 activity. The increase of [Ca^2+^] has been postulated to be associated with glutamate-induced cell death. In cultured hippocampal neurons exposed to 125 μM glutamate, [Ca^2+^] was promptly elevated, and then leveled off with significantly higher values compared to that for control group throughout a recording period of 15 min. In contrast, [Ca^2+^] in cultured hippocampal neurons pretreated with COS (1.0 mg/mL) showed significantly lower values than those for cultured hippocampal neurons without COS pretreatment over a recording period of 15 min, suggesting that COS pretreatment significantly inhibited the [Ca^2+^] increase. Since glutamate-evoked cell injury in hippocampal neurons is involved in many CNS disorders, their study may raise the possibility of developing COS as a potential agent for the prevention and treatment of some CNS diseases.

Apoptosis inhibition activity of chitosan and COS might be extended to the intervention of neurodegenerative disorders. However, further studies, such as *in vivo* tests, are needed to clarify the neuroprotective properties of chitosan and COS.

## 6. Other Activities

The possible role of chitosan in preventing oxidative stress-induced by amyloid β formation in NT2 neuron cells was investigated by Khodagholi *et al*. [54]. On the other hand, the evaluation utility of chitosan nanoparticles with an *in-vitro* model of acrolein-mediated cell injury using PC-12 cells was investigated by Cho *et al*. [71]. The particles effectively, and statistically, reduced damage to membrane integrity, secondary oxidative stress, and lipid peroxidation. Their study suggests that a chitosan nanoparticle-based therapy to interfere with “secondary” injury is possible.

Moreover, the development of an effective delivery system is needed to provide sufficient drug concentration into the brain to prevent cell death. Using avidin (SA)-biotin (BIO) technology, Aktas *et al.* [72] describe the design of chitosan (CS) nanospheres conjugated with poly(ethylene glycol) (PEG) bearing the OX26 monoclonal antibody whose affinity for the transferrin receptor (TfR) may trigger receptor mediated transport across blood-brain barrier. Their findings indicate that this novel targeted nanoparticulate drug delivery system was able to translocate into the brain tissue after intravenously administration. Consequently, chitosan is a promising carrier for the transport of the anticaspase peptide Z-DEVD-FMK into the brain.

## 7. Conclusions

In recent years, the marine environment has shown to provide extremely rich biological active compounds. Chitosan, one of the bioactive compounds derived from the sea, has been shown to possess many biological activities. In fact, the interest in chitosan and its derivatives for the treatment of neurological disorders appear to be an emerging field. Some of the representative’s examples are presented in this review with a focus on neuroprotective properties of chitosan and its derivatives. According to presented data, it seems that chitosan and its derivatives are promising neuroprotective agents, as they showed neuroprotective properties such as: suppression of β-amyloid formation, AChEIs, anti-neuroinflammatory activity, apoptosis inhibitors, *etc.* Up until now, most neuroprotective activities of chitosan and its derivatives have been observed *in vitro*. Therefore, further studies are needed in order to investigate their activity in mouse model systems and/or human subjects. In conclusion, these results reveal the potential of chitosan and its derivatives as potential therapeutic candidates for neurodegenerative disorder and their involvement in the future pharmaceuticals are promising.

## Figures and Tables

**Table 1:** Cholinesterase inhibitory activities of COS derivatives.

COS	IC_50_	Ref
90-HMWCOS	2.59 mg/mL	[60]
90-MMWCOS	1.67 mg/mL	[60]
90-LMWCOS	3.52 mg/mL	[60]
50-HMWCOS	1.98 mg/mL	[60]
50-MMWCOS	2.93 mg/mL	[60]
50-LMWCOS	>4.00 mg/mL	[60]
AE-COS	56.5 ± 0.26 μg/mL	[61]
DMAE-COS	24.1 ± 0.39 μg/mL	[61]
DEAE-COS	9.2 ± 0.33 μg/mL	[61]
Eserine^a^	0.0089 ± 0.00005 μg/mL	[61]

a positive control.
